# Artificial intelligence-enhanced three-dimensional echocardiography reveals left atrial-ventricular coupling index as a novel prognostic marker in coronary artery disease

**DOI:** 10.1186/s12872-025-05341-z

**Published:** 2025-12-03

**Authors:** Siwen Qin, Difei Li, Qin Feng, Yanchao Zhang, Donghui Zhou, Shuang Liu

**Affiliations:** 1 Department of Ultrasound, The Fourth Affiliated Hospital of China Medical University, Shenyang, 110000 People’s Republic of China; 2 Department of Cardiovascular Medicine, The Fourth Affiliated Hospital of China Medical University, Shenyang, 110000 People’s Republic of China

**Keywords:** Left atrial-ventricular coupling index, Coronary artery disease, AI-enhanced 3D echocardiography, Major adverse cardiovascular events, Gensini scores, Cardiovascular risk assessment

## Abstract

**Background:**

The Left Atrial-Ventricular Coupling Index (LACI), which quantifies the volumetric interaction between the left atrium and ventricle, has emerged as a promising prognostic marker for cardiovascular events.

**Methods:**

We applied three-dimensional echocardiography (3DE) and AI quantitative software to automatically obtain the new echocardiographic parameter LACI in 1084 coronary artery disease (CAD) patients at the Fourth Affiliated Hospital of China Medical University. LACI was defined as the ratio between the left atrial end-diastolic volume and the left ventricular end-diastolic volume. The primary endpoints were major adverse cardiovascular events (MACE), including unstable angina, myocardial reinfarction, heart failure (HF) and death.

**Results:**

Over a median follow-up of 13 months, 216 MACE were recorded. LACI was significantly higher in patients with MACE than in those without (0.22 [IQR 0.17–0.28] vs. 0.20 [IQR 0.15–0.25], *p* < 0.001). ROC analysis indicated that LACI demonstrated higher predictive accuracy for MACE than traditional echocardiography parameters. Multivariable Cox regression analysis revealed that LACI was significantly correlated with MACE (HR: 1.68, 95% CI [1.25–2.27], *p* < 0.001), especially in patients with severe lesion subgroups according to the Gensini scores (HR 1.33, 95% CI [1.17–1.52], *p* < 0.002) and male (HR: 1.37, 95% CI [1.20–1.56], *p* < 0.002). Furthermore, LACI assessment enabled further risk stratification in high-risk patients with severe lesion (*p* < 0.001 on log-rank testing). Consistently, category-free NRI and IDI confirmed the improvement by LACI to stratify MACE risk in severe lesion patients (*p* < 0.05).

**Conclusion:**

Our findings demonstrated that AI-enhanced 3DE-derived LACI provided incremental prognostic value beyond conventional echocardiographic parameters in assessing the prognosis among CAD patients, especially in severe coronary lesion subgroups.

**Supplementary Information:**

The online version contains supplementary material available at 10.1186/s12872-025-05341-z.

## Introduction

Coronary artery disease (CAD) remains a major global health challenge [1, 2], characterized by the narrowing or blockage of coronary arteries primarily due to atherosclerosis. This condition leads to the occurrence of major adverse cardiovascular events (MACE), consequently affecting quality of life and mortality rates [3, 4]. Recent epidemiological studies indicated that CAD gave rise to more than 7 million deaths annually [5]. At the same time, CAD imposed a considerable economic burden on healthcare systems as well [6]. Therefore, prognostic risk stratification of patients with CAD for the implementation of early interventions is essential to reduce the burden of disease.

Various imaging techniques are currently used in clinical settings to assess cardiac function for early risk stratification and intervention. Cardiac magnetic resonance (CMR) imaging is widely considered as the gold standard for evaluating cardiac function. However, CMR is not always readily available, especially in remote or economically disadvantaged areas [7]. In comparison, echocardiography has significant advantages due to its low price and non-invasive nature, real-time imaging capabilities, and lack of ionizing radiation.

At the same time, several studies have demonstrated that three-dimensional echocardiography (3DE) is more accurate in the evaluation of cardiac chamber volume [8, 9]. And artificial intelligence (AI) has been developed for echocardiography to improve efficacy and efficiency in assessing cardiac function [10]. De Alexandria AR et al. applied AI in echocardiography, focusing on automated left ventricular segmentation using deep learning-based semantic segmentation algorithms [11]. Current research has extended the application of deep learning models to automate the analysis of point-of-care ultrasound (POCUS) examinations across multi-disease datasets [12].

Advanced imaging techniques have increased awareness that many cardiovascular events are significantly influenced by the functions of the left ventricle (LV) and left atrium (LA) [13]. Previous studies had used LA or LV related parameters as markers for assessment of cardiac performance for the prediction of cardiovascular events [14–16]. On this basis, it has been suggested that a novel parameter, which considers simultaneously LA and LV, may be a better predictor than individual LA or LV parameters [17, 18].

The hemodynamic basis for this interdependence lies in diastolic mechanisms: early diastolic filling generates a vortex, enhancing kinetic energy and LV expansion, while diastasis optimizes preload via cardiomyocyte stretching [17]. These mechanisms highlight LA-LV interdependence, justifying LACI ’s prognostic value—a combined measure of LA and LV end-diastolic volumes [19–22]. At the same time, Federico Fortuni et al. linked LACI to heart failure via LV diastolic dysfunction [18], and Theo Pezel et al. validated its prediction of heart failure, (HF), atrial fibrillation (AF), and MACE [14], CAD applications remain scarce. In addition, previous study indicated that a high Gensini score was also a risk factor for patients with CAD and was associated with a high incidence of MACE [23, 24].

This study aims to investigate the prognostic value of LACI, derived from AI-enhanced 3DE quantitative software Dynamic Heart Model (DHM), for risk stratification in patients with CAD for the clinic to implement early intervention in high-risk patients.

## Methods

### Study design and population

This study was a retrospective cohort study that included 1084 patients older than 18 years old who met relevant clinical criteria and had diagnosed with CAD at the Fourth Affiliated Hospital of China Medical University from February 2022 to December 2023 and who agreed to participate in the study. CAD was defined as ≥ 50% stenosis by quantitative invasive coronary angiography (ICA). All patients underwent ICA and had >50% stenosis [25].

### Follow-up and endpoints

They were followed up for the occurrence of MACE, which served as the primary endpoint. The MACE was defined as a composite of death, hospital readmission for unstable angina, myocardial reinfarction, and HF [26]. If a patient had multiple MACE during the study, the priority was assigned as follows: death >reinfarction >HF >unstable angina. Furthermore, only one MACE event was counted per patient. The last date for the period was June 30, 2024.

### Medication definitions

Hypertensive medication was defined as the use of diuretics, angiotensin-converting enzyme inhibitors (ACEIs), angiotensin receptor blockers (ARBs), β-blockers, calcium channel blockers (CCBs), or mineralocorticoid receptor antagonists (MRAs) for ≥ 30 days at baseline. Lipid-lowering therapy was defined as the administration of statins, ezetimibe, or fibrates for ≥ 4 weeks prior to baseline assessment. The diagnosis of left ventricular diastolic dysfunction (LVDD) was established with reference to relevant echocardiographic parameters [27].

### Exclusion criteria

**Fig. 1:**
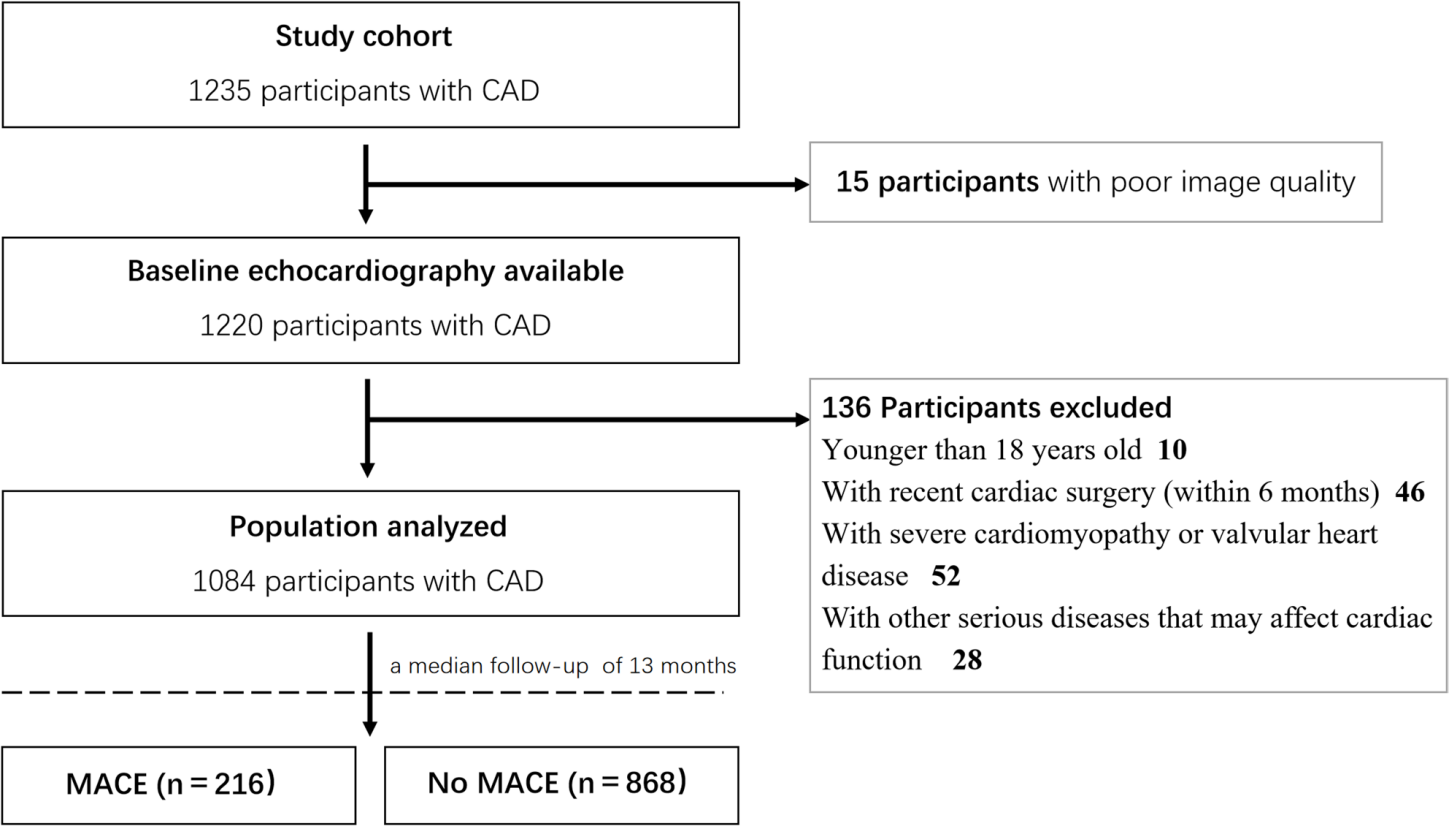
Flowchart of the study. CAD, coronary artery diseases; MACE, major adverse cardiovascular events

Patients with recent cardiac surgery (within 6 months), severe cardiomyopathy, valvular heart disease or other serious diseases that may affect cardiac function (e. hepatic vs. renal failure), unable to complete echocardiography, and younger than 18 years old were excluded (Fig. 1). The collected data included basic information of patients, including age, sex, medical history, echocardiographic results. The study was approved by the ethics committee of the Fourth Affiliated Hospital of China Medical University (EC-2024-KS-117).

### Echocardiographic analysis

A Philips EPIQ CVx diagnostic ultrasound machine equipped with an S5-1 phased array probe (frequency 1–5 MHz) and an X5-1 matrix probe (frequency 1–5 MHz) was used. Echocardiographic examination and image acquisition were performed according to the guidelines established by the Echocardiography Group of the Chinese Medical Association’s Ultrasound Medical Branch and the American Society of Echocardiography (ASE), and all the images were stored in the original DICOM format and were captured by senior ultrasonographers. The standard duration for echocardiographic data acquisitions was 3 to 5 cardiac cycles (extended to five cardiac cycles in patients with atrial fibrillation [28]).

### AI-powered analysis

The DHM is an AI-powered quantitative analysis software integrated with Philips 3D echocardiography systems. Its operational workflow begins with ECG-gated acquisition of apical 4-chamber views over 3–5 cardiac cycles using 3D echocardiography to ensure temporal synchronization. The LV and LA regions were manually traced by the operator. The system then employs deep learning algorithms to automatically segment and calculate both left ventricular end-diastolic volume (LVEDV) and left atrial end-diastolic volume (LAEDV) by analyzing 3D spatiotemporal data.

This process is typically completed in under one minute with high reproducibility, significantly reducing inter-observer variability compared to traditional manual 2D tracing methods. However, DHM ‘s accuracy relies heavily on high-quality 3DE images. Poor acoustic windows or suboptimal patient conditions (e.g., obesity, lung disease) may compromise measurements. Therefore, patients with poor image quality were excluded from our analysis.

### Left atrial-ventricular coupling index

**Fig. 2:**
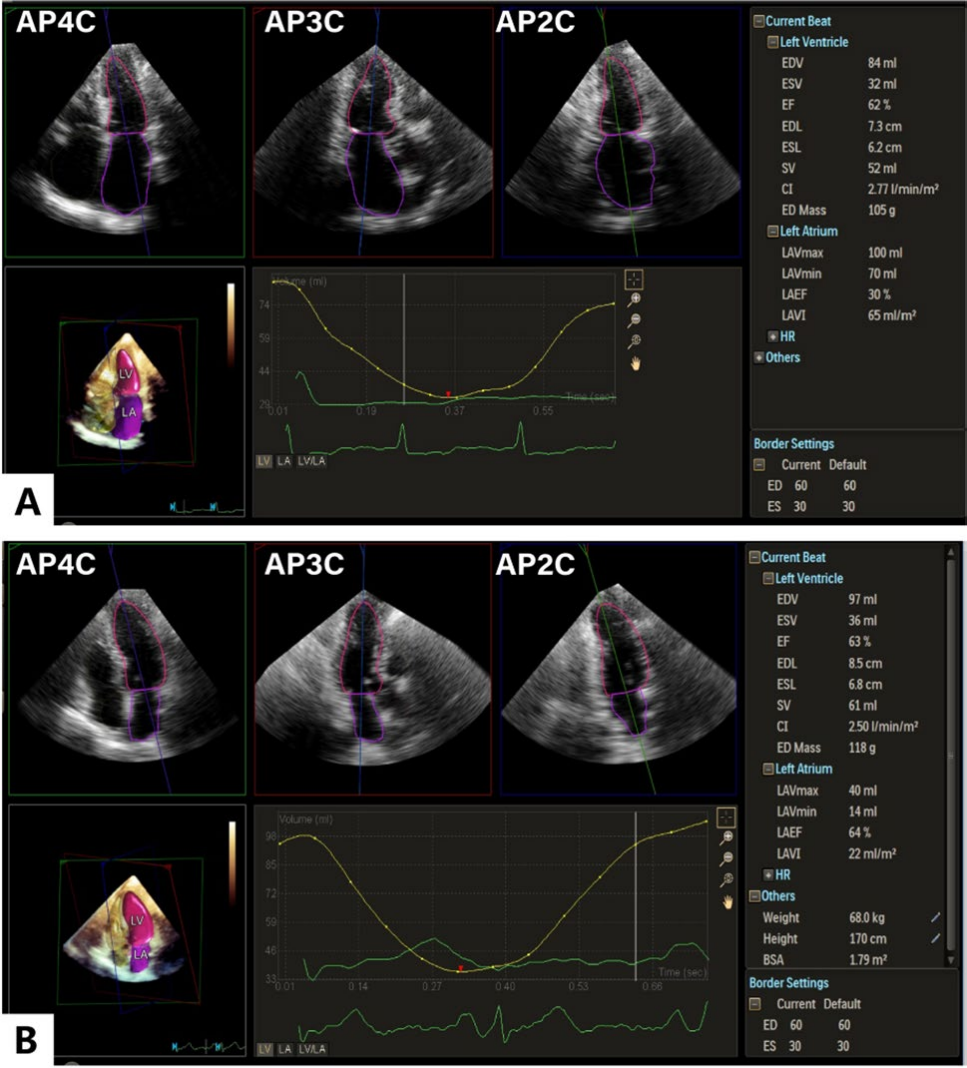
Left atrial ventricular coupling index (LACI). Using three-dimensional echocardiography and AI intelligent quantitative software Dynamic Heart Model (DHM) automatically obtained the new echocardiographic parameter left atrial ventricular coupling index (LACI). **A**,** B** 2D apical four-, three-, and two-chamber views separately extracted from coronary artery diseases (CAD) patients with heart failure and without major adverse cardiac event (MACE) at end-diastole. Red and purple lines denote LV and LA endocardial borders, respectively, registered using fully automated software. AP2(3,4)C, apical two-(three-, four-)chamber view; CI, cardiac index; ED(S)L, end-diastolic (end-systolic) length; ed(es)LVLAVR, left ventricular-left atrial volume ratio at LV end-diastole (end-systole); ED(S)V, end-diastolic (end-systolic) volume; EF, ejection fraction; LA, left atrium; LAEF, left atrial ejection fraction; LAVI, left atrial volume index; LAVmax, maximal left atrial volume; LAVmin, minimal left atrial volume; LV, left ventricular; LVEDV, left ventricular end-diastolic volume; LVEF, left ventricular ejection fraction; LVESV, left ventricular end-systolic volume; SV, stroke volume

LACI was calculated as the ratio between LAEDV and LVEDV measured by echocardiography. Both LA and LV volumes are measured in the same end-diastolic phase. EDV was evaluated by 3DE (Fig. 2). 3DE utilizes matrix-array transducers to acquire volumetric heart data, which are then reconstructed into dynamic 3D images through spatial beamforming and voxel-based rendering, enabling accurate cardiac structural and functional assessment beyond conventional 2D limitations [29]. LACI values are expressed as a percentage. A higher LACI reflects a greater imbalance between left atrial and left ventricular volumes during ventricular end-diastole, suggesting greater impairment of left atrial ventricular coupling.

### Gensini scores

The Gensini scores were calculated as follows: segmentation of diseased vessels: The diseased vessels were divided into the left main stem, left anterior descending branch, circumflex branch and right coronary artery. Assessment of stenosis: The degree of lesion of each vessel was quantitatively assessed, and the degree of stenosis was standardized at the most severe point. A stenosis diameter of ≤ 25% was scored as 1 point, 26%−50% as 2 points, 51%−75% as 4 points, 76%−90% as 8 points, 91%−99% as 16 points, and 100% as 32 points. Different segments of coronary arteries were multiplied by the appropriate coefficients. Calculation of the total score: the final score is the sum of the score of each branch [30].

According to the Gensini scoring system, all the patients were stratified into mild lesion group (≤ 20 points), moderate lesion group (21–40 points), severe lesion group(>40 points).

### Statistical analysis

Statistical analysis was performed using R software (version 4.2.2) along with MSTATA software (www.mstata.com). Data were summarized as mean (standard deviation) to represent normally distributed variables, median (interquartile range [IQR]) to represent non-normally distributed variables, and categorical variables were expressed as frequencies and percentages. Correlation was assessed by Spearman rank correlation coefficients. Continuous datasets were compared with the nonparametric Mann-Whitney U test. The optimal dichotomous cut value of the LACI was determined by the Youden index.

The Kaplan-Meier method was used to analyze the occurrence of MACE, and differences between groups were evaluated with the log-rank test. Restricted Cubic Splines (RCS) analysis with 3 knots (placed at the 10th, 50th, and 90th percentiles of LACI) was performed to evaluate the nonlinear association between MACE and LACI among CAD patients. Receiver operating characteristic (ROC) curve analysis was performed to compare the predictive performance of LACI and conventional echocardiography parameters for MACE. The DeLong test was employed to statistically compare the differences in area under the curve (AUC) between different predictive models. Cox proportional risk regression models were used to calculate univariate hazard ratios (HR) in the assessment of MACE. Variables associated with the outcome (*p* < 0.05) were subsequently incorporated into a multivariable Cox proportional hazards model (Supplementary Table 1). Utilizing a quasi-Bayesian causal mediation analysis, this study investigated the mediating effect of LVDD on the association between LACI and MACE.

Subgroup analyses were conducted based on Gensini scores, gender and age group. To address potential type I error inflation due to multiple comparisons, Bonferroni correction was applied for both interaction *P*-values and within-subgroup *P*-values. Exploratory subgroup results were interpreted with the implementation of Bonferroni correction. To assess the reclassification improvement of LACI in risk prediction models that included the Gensini scores, net reclassification improvement (NRI) and integrated classification improvement (IDI) measures were used. Due to multicollinearity, only left ventricular ejection fraction (LVEF), left atrial ejection fraction (LAEF), left ventricular end-diastolic volume (LVEDV) were included as echocardiography parameters in the model construction (Supplementary Table 2). In addition, reproducibility testing was performed on 150 randomly selected cases (50 with mild and 50 with moderate and 50 with severe Gensini scores). To assess consistency, we performed an intergroup test using the correlation coefficient and an intragroup test utilizing the Intraclass Correlation Coefficient (ICC).

## Results

### Study cohort

The study cohort comprised 1,084 patients with a mean age of 63.55 ± 10.55 years, including 752 males (69%). Comorbidities included hypertension (*n* = 706), diabetes mellitus (DM, *n* = 391), atrial fibrillation (AF, *n* = 57), prior myocardial infarction (MI, *n* = 306), and prior percutaneous coronary intervention (PCI, *n* = 342) (Table 1).

**Table 1:** Population characteristics of participants at baseline

Characteristic	MACE		*P*-value
	no, *N* = 868	yes, *N* = 216	
Sex (male)	606 (69.8%)	146 (67.6%)	0.526
Age group			0.214
[26,51.2)	99 (11.4%)	27 (12.5%)	
[51.2,63.3)	316 (36.4%)	63 (29.2%)	
[63.3,74.2)	344 (39.6%)	92 (42.6%)	
[74.2,90]	109 (12.6%)	34 (15.7%)	
BMI, kg/m²	23.53 (21.71, 25.95)	23.44 (21.71, 25.86)	0.613
BSA, m²	1.79 (1.67, 1.91)	1.79 (1.64, 1.91)	0.843
SBP, mmHg	134 (120, 147)	138 (122, 145)	0.941
DBP, mmHg	80 (74, 88)	80 (70, 87)	0.080
Previous smoke, N%	418 (48.2%)	108 (50.0%)	0.628
Previous drink, N%	140 (16.1%)	36 (16.7%)	0.848
Hypertension, N%	550 (63.4%)	156 (72.2%)	0.015
DM, N%	293 (33.8%)	98 (45.4%)	0.001
AF, N%	41 (4.7%)	16 (7.4%)	0.114
Previous MI, N%	236 (27.2%)	70 (32.4%)	0.127
Previous PCI, N%	252 (29.0%)	90 (41.7%)	< 0.001
Gensini group, N%			< 0.001
Mild lesion	99 (11.4%)	12 (5.6%)	
Moderate lesion	345 (39.7%)	67 (31.0%)	
Severe lesion	424 (48.8%)	137 (63.4%)	
LVDD, n (%)			0.003
0	543 (62.6%)	111 (51.4%)	
1	325 (37.4%)	105 (48.6%)	
Lipid-lowering medication, N%	72 (8.3%)	20 (9.2%)	0.115
WBC, 10^9/L	7.18 (6.10, 8.58)	7.31 (6.30, 8.40)	0.341
PLT, 10^9/L	194 (160, 234)	204 (167, 236)	0.209
Hb, g/L	131 ± 16	129 ± 16	0.022
ALB, g/L	39.4 (37.3, 42.0)	38.8 (36.8, 41.2)	0.022
CHOL, mmol/L	3.54 (2.80, 4.34)	3.29 (2.81, 3.90)	0.012
HDL, mmol/L	0.85 (0.73, 1.02)	0.82 (0.69, 0.95)	0.014
ApoA, g/L	1.04 (0.95, 1.15)	1.02 (0.92, 1.11)	0.041
ApoB, g/L	0.67 (0.51, 0.84)	0.63 (0.52, 0.71)	0.008
NT-proBNP, pg/ml	300 (98, 900)	589 (173, 1,563)	< 0.001

*MACE* major adverse cardiovascular events, *BMI* body mass index, *BSA* body surface area, *SBP* systolic blood pressure, *DBP* diastolic blood pressure, *DM* diabetes mellitus, *AF* atrial fibrillation, *MI* myocardial infarction, *PCI* percutaneous coronary intervention, *LVDD* left ventricular diastolic dysfunction, *WBC* white blood cell counts, *PLT* platelet, *Hb* hemoglobin, *ALB* albumin, *CHOL* total cholesterol, *HDL* high-density lipoprotein, *NT-proBNP* N-terminal pro-B-type natriuretic peptide

During a median follow-up of 13 months (IQR: 8–22), 216 MACE events occurred, consisting of 21 deaths, 19 reinfarctions, 48 heart failure, and 128 unstable anginas. Stratified by Gensini scores, the cohort included 111 mild (≤ 20 points), 412 moderate (21–40 points), and 561 severe (> 40 points) lesion cases (Table 1).

Upon analyzing the baseline characteristics, there were no significant differences (*p* > 0.05) in demographic and clinical variables such as gender distribution, age group, body mass index (BMI), systolic blood pressure (SBP), diastolic blood pressure (DBP), smoking and drinking habits, AF and history of MI between the groups based on MACE occurrence. However, individuals who experienced MACE had a higher incidence of hypertension (72.2% vs. 63.4%, *p* = 0.015), DM (45.4% vs. 33.8%, *p* = 0.001), and previous PCI (41.7% vs. 29.0%, *p* < 0.001) compared to those who did not experience MACE. Furthermore, participants who experienced MACE had a higher proportion in the severe lesion of Gensini scores (63.4% vs. 48.8%), indicating a higher risk for MACE events in this group.

### LACI distribution and relationship to each LA and LV parameters

In the overall cohort, the median LACI was 0.20 [IQR 0.16–0.26], with significantly higher in patients who developed MACE than in those without MACE (0.22 [IQR 0.17–0.28] vs. 0.20 [IQR 0.15–0.25], *p* < 0.001). Regarding the definition of LACI, it was correlated with other echocardiographic parameters (Supplementary Table 3). Notably, LACI showed significant negative correlation with LA other parameters such as LAEF (*r* = −0.62, *p* < 0.001), but no significant association with LV other function parameters such as LVESV. A detailed overview of the echocardiographic parameters is shown in Table 2.

**Table 2:** Population characteristics of participants in the echocardiography

Characteristic	MACE		*P*-value
	no, *N* = 868	yes, *N* = 216	
LAD-2D, mm	38.0 (36.0, 41.0)	39.0 (36.0, 42.0)	0.021
LVDd-2D, mm	48.0 (45.0, 52.0)	49.5 (46.0, 53.0)	0.002
LVDs-2D, mm	31 (28, 35)	33 (28, 38)	0.002
RVOTD-2D, mm	27.00 (25.00, 29.00)	26.00 (25.00, 29.00)	0.331
MVE, m/s	0.80 (0.60, 0.90)	0.80 (0.60, 1.00)	0.139
MVA, m/s	0.90 (0.80, 1.10)	0.90 (0.80, 1.00)	0.793
Sep e’, cm/s	6.00 (5.00, 8.00)	6.00 (5.00, 7.07)	0.296
Lat e’, cm/s	8.00 (7.00, 10.00)	9.00 (7.00, 10.00)	0.818
E/A-2D	0.80 (0.70, 1.05)	0.80 (0.70, 1.10)	0.165
E/e’−2D	10.0 (8.0, 12.0)	10.0 (9.0, 14.0)	0.046
LAVmin-3D, ml	23 (17, 31)	26 (20, 37)	< 0.001
LACI	0.20 (0.15, 0.25)	0.22 (0.17, 0.28)	0.001
LAVI-3D, ml/m²	33 (27, 40)	34 (28, 42)	0.202
LAVmax-3D, ml	59 (48, 72)	61 (49, 74)	0.244
LAEF-3D, %	60 (53, 66)	56 (47, 63)	< 0.001
LVEDmass-3D, g	138 (120, 162)	141 (121, 168)	0.263
LVEDV-3D, ml	116 (97, 142)	120 (100, 151)	0.036
LVESV-3D, ml	46 (36, 59)	47 (38, 67)	0.014
LVEF-3D, %	60 (55, 64)	58 (53, 63)	0.006

All continuous parameters were standardized

*2D* two-dimensional, *LAD* left atrial diameter, *LVDd* left ventricular end-diastolic dimension, *LVDs* left ventricular end-systolic dimension, *RVOTD* right ventricular outflow tract diameter, *MVE* mitral valve E velocity, *MVA* mitral valve A velocity, *E/A* ratio of early to late diastolic peak velocities (or E-wave to A-wave ratio), *e’* early diastolic velocity of mitral annulus, *Sep e’* septal e’ velocity, *Lat e’* lateral e’ velocity, *LAVmin* minimal left atrial volume, *LACI* left atrial-ventricular coupling index, *LVEDmass* left ventricular end-diastolic mass, *LAVmax* maximal left atrial volume, *LAEF* left atrial ejection fraction, *LAVI* left atrial volume index, *LVEDV* left ventricular end-diastolic volume, *LVESV* left ventricular end-systolic volume, *LVEF* left ventricular ejection fraction

### The relationship between LACI and MACE

**Fig. 3:**
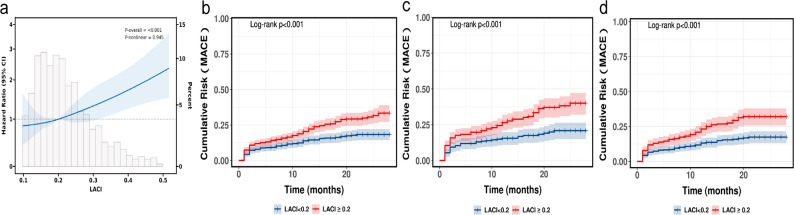
Association between LACI and MACE with the RCS (**a**), Kaplan-Meier curves illustrating LACI stratification regarding the prognosis for all CAD patients (**b**), Gensini scores severe lesion group (**c**) and males (**d**). **a** Model with 3 knots located at 10th, 50th and 90th percentiles. Y-axis represents the HR to present MACE incidence for any value of LACI compared to individuals with reference value (50th percentile) of LACI. **b** The Kaplan-Meier survival curves for MACE stratified by the Youden index of LACI. **c** The Kaplan-Meier curves for MACE stratified by the Youden index of LACI in severe lesion subgroups according to the Gensini scores. **d** The Kaplan-Meier curves for MACE stratified by the Youden index of LACI in males

**Fig. 4:**
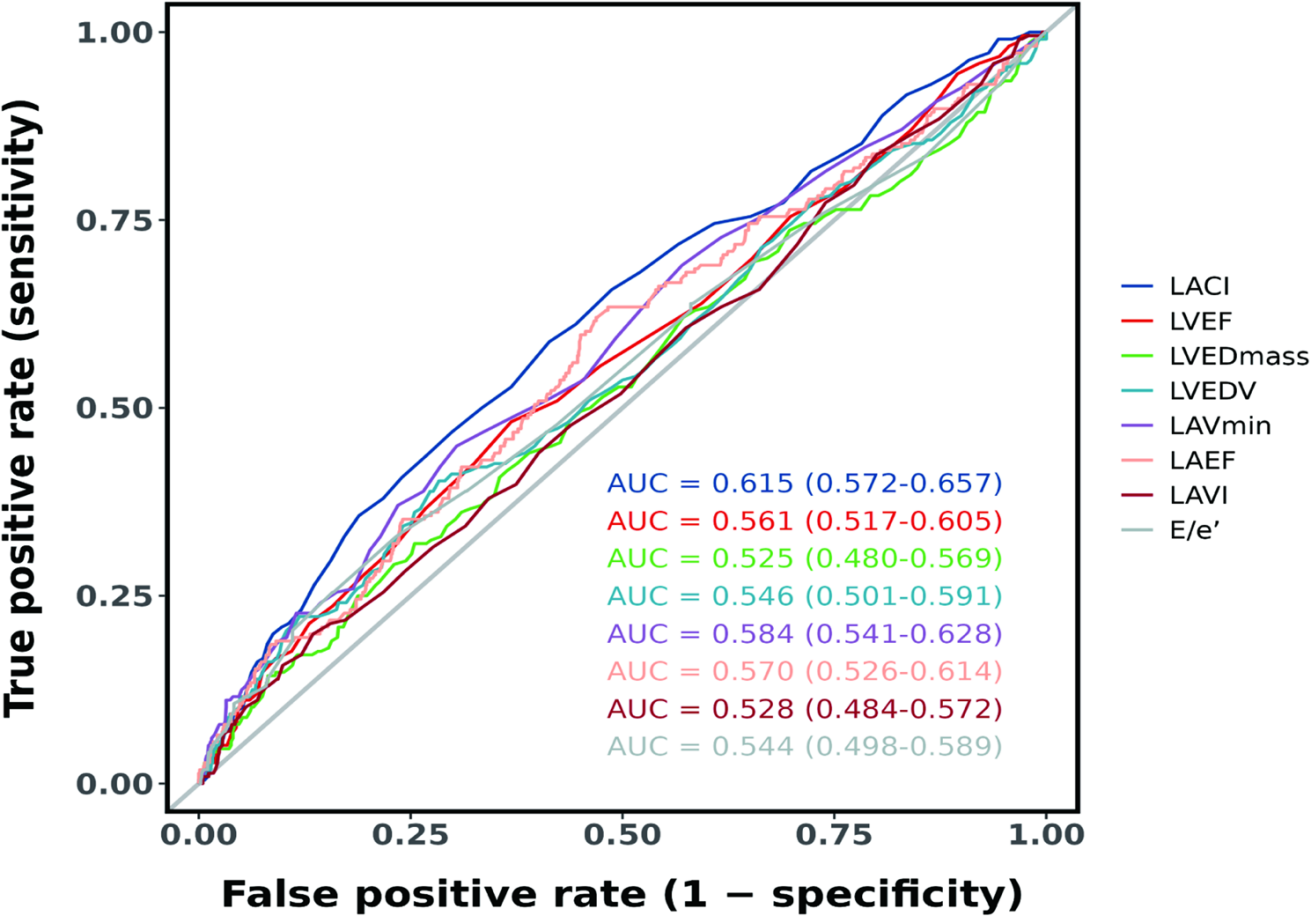
ROC analysis for the predictive accuracy of LACI for MACE compared to traditional echocardiography parameters. LACI, left atrial-ventricular coupling index; LVEDmass, left ventricular end-diastolic mass; LAVmin, minimal left atrial volume; LAEF, left atrial ejection fraction; LAVI, left atrial volume index; LVEDV, left ventricular end-diastolic volume; LVEF, left ventricular ejection fraction

RCS indicated a steady increase in the risk of MACE as LACI values rose, demonstrating an almost linear trend (Fig. 3a). ROC analysis demonstrated that LACI ‘s superior predictive accuracy for MACE than traditional echocardiography parameters (Fig. 4). The results of unadjusted and adjusted Cox proportional hazard models for LACI and the main LA and LV echocardiography parameters are indicated in Table 3. LACI was positively associated with MACE after adjustment for baseline confounders including age, sex, BMI, hypertension, DM, previous PCI, Gensini scores group, white blood cell count (WBC), hemoglobin, Albumin, Apolipoprotein A (ApoA), Total cholesterol (CHOL), N-terminal pro-B-type natriuretic peptide (NT-proBNP) (adjusted hazard ratio [HR], 1.68, 95% CI [1.25–2.27], *P* < 0.001). Using the Youden index-derived cutoff (LACI = 0.20), Kaplan-Meier analysis showed significantly worse outcomes in high-LACI patients (log-rank p < 0.001; Fig. 3b). In the unadjusted model, the total effect, direct effect, and indirect effect of LACI on MACE through LVDD were all statistically significant (Supplementary Fig. 1; Supplementary Table 4). The mediation proportion attributable to LVDD accounted for 18.0% (95% CI: 3.2 to 46.0) of the total effect. In the adjusted model, the total effect (β = −56.92, p = 0.004) and direct effect (β = −50.46, p < 0.001) of LACI on MACE remained significant. In contrast, the indirect effect mediated through LVDD was no longer significant (β = −6.46, p = 0.484).

**Table 3:** Univariate and multivariable analyses comparing conventional echocardiography parameters and LACI for MACE prediction

Characteristic	Model 1				Model 2		
	HR	95% CI	*P*-value		HR	95% CI	*P*-value
LACI							
< 0.2	—	—			—	—	
≥ 0.2	**1.72**	1.30, 2.27	< 0.001		1.68	1.25, 2.27	< 0.001
LACI (continuous)	**1.27**	1.14, 1.42	< 0.001		1.49	1.28, 1.74	< 0.001
E/e’	1.08	1.04, 1.13	< 0.001		1.02	0.97, 1.07	0.390
LAVI	1.12	0.99, 1.27	0.066		1.09	0.95, 1.24	0.230
LAVmin	1.30	1.17, 1.44	< 0.001		1.25	1.12, 1.39	< 0.001
LAEF	0.72	0.64, 0.81	< 0.001		0.74	0.66, 0.84	< 0.001
LVEDV	1.19	1.06, 1.34	0.003		1.16	1.03, 1.32	0.018
LVEF	0.81	0.71, 0.91	< 0.001		0.85	0.74, 0.97	0.014
LVEDmass	1.13	1.00, 1.29	0.058		1.11	0.96, 1.29	0.165

Model 1: no covariables adjusted

Model 2: age, sex, BMI, hypertension, DM, previous PCI, Gensini scores group, WBC, hemoglobin, ALB, ApoA, CHOL, NT-proBNP

*HR* Hazard Ratio, *CI* Confidence Interval, *LACI* left atrial-ventricular coupling index, *LVEDmass* left ventricular end-diastolic mass, *LAVmin* minimal left atrial volume, *LAEF* left atrial ejection fraction, *LAVI* left atrial volume index, *LVEDV* left ventricular end-diastolic volume, *LVEF* left ventricular ejection fraction

### Subgroup analysis

In the severe Gensini scores subgroup, LACI showed particularly robust association with MACE (HR 1.33, 95% CI [1.17–1.52], *p* < 0.002) (Table 4). The use of the LACI assessment of the optimal cut-off value determined by the Youden index enabled further risk stratification among severe lesion CAD patients according to Gensini scores (log-rank test *p* < 0.001) (Fig. 3c). In addition, on classification by gender, LACI was significantly associated with MACE in male patients, but did not show a significant association with MACE in female patients (male: HR 1.37, 95% CI [1.20–1.56], *p* < 0.002; female: HR 1.13, 95% CI [0.92–1.38], *p* = 0.486) (Fig. 3d) (Table 4).

**Table 4:** Subgroup analysis of the association between LACI and MACE risk

Subgroup	HR (95% CI)	*P* value (Bonferroni corrected *P*)	*P* for interaction (Bonferroni corrected *P*)
Overall	1.27 (1.14–1.42)	< 0.001	
Sex			0.11(0.33)
Male	1.37 (1.20–1.56)	< 0.001( <0.002)	
Female	1.13 (0.92–1.38)	0.243(0.486)	
Age group			0.408(1.0)
[26,51.2)	1.47 (1.04–2.07)	0.029(0.116)	
[51.2,63.3)	1.44 (1.01–2.07)	0.047(0.188)	
[63.3,74.2)	1.16 (0.97–1.39)	0.111(0.444)	
[74.2,90]	1.39 ( 1.12–1.72)	0.002 (0.008)	
Gensini group			0.351(1.0)
Mild and moderate lesion	1.20 (0.99–1.46)	0.064(0.128)	
Severe lesion	1.33 (1.17–1.52)	< 0.001( <0.002)	

*HR* hazard ratios. CI Confidence Interval, *MACE* major adverse cardiovascular events

**Fig. 5:**
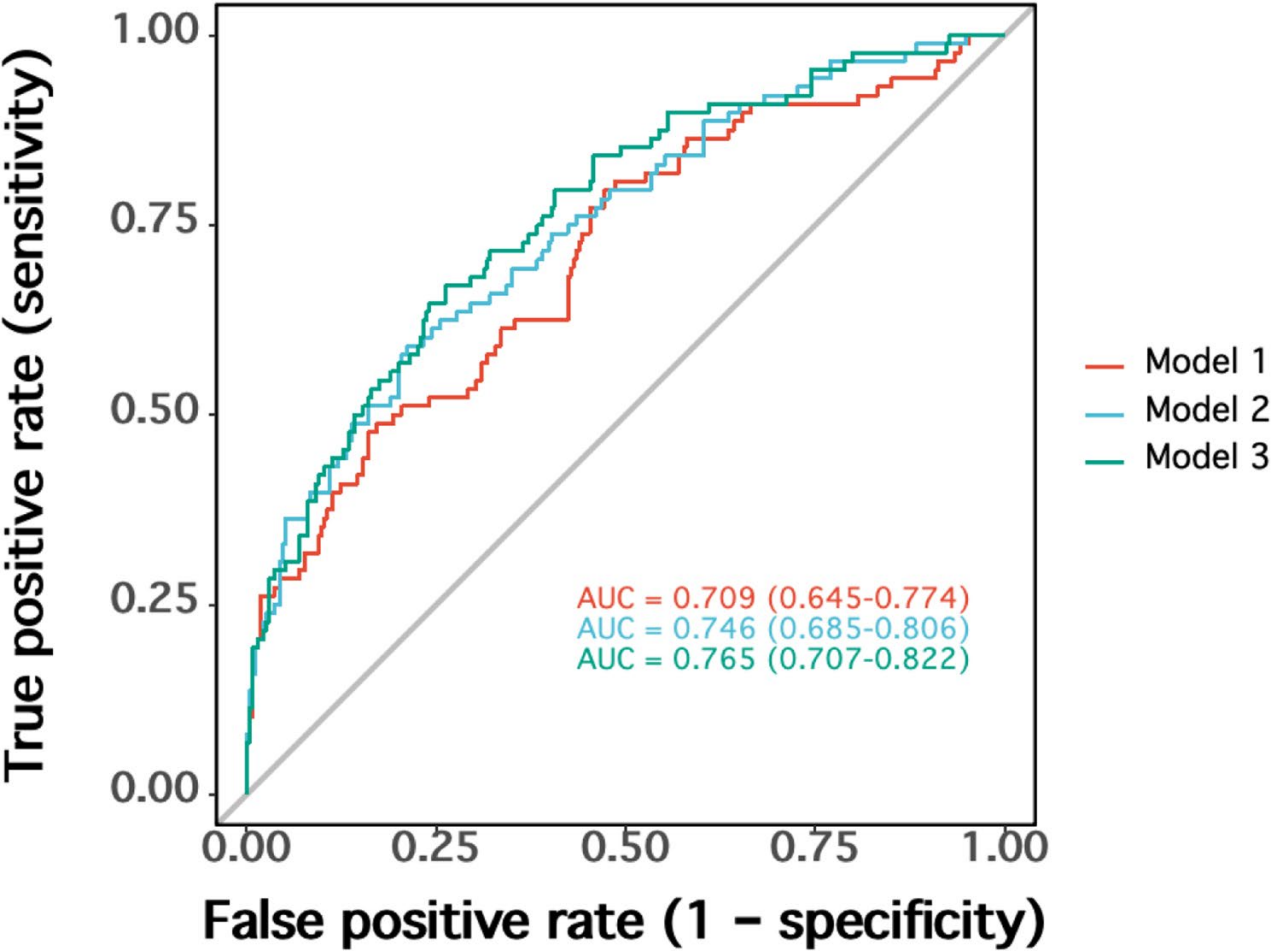
ROC analysis for incremental predictive value of LACI in the severe lesion group according to Gensini scores. Model 1: Hypertension, DM, Previous PCI, Gensini scores group, WBC, Hb, ALB, ApoA, CHOL, NT-proBNP. Model 2: Model 1 + LVEF, LVEDV, LAEF. Model 3: Model 2 + LACI

The multivariable model with LACI showed significant improvement to model discrimination and reclassification compared to clinical model with traditional clinical risk factors and conventional echocardiography parameters to predict MACE in the Gensini scores severe lesion group (AUC = 0.765, 95% CI [0.707–0.822]) (Fig. 5) (Table 5). Assessing the prognostic improvement in discrimination of different models, the addition of LACI to Gensini scores resulted in significant improvement, the NRI reached 0.400 (95% CI [0.243–0.709], *p* < 0.001) with an IDI of 0.052 (95% CI [0.029–0.074], *p* < 0.001). However, the generalizability of these subgroup-specific improvements requires validation in independent cohorts, particularly given the exploratory design of these analyses.

**Table 5:** Incremental predictive value of LACI in the Gensini score severe lesion group

Outcome	Model	AUC 95% CI	Delong Test		NRI		IDI	
			Z score	*P*-value	95% CI	*P*-value	95% CI	*P*-value
MACE	Model 1	0.709(0.645–0.774)	Reference	-	Reference	-	Reference	-
MACE	Model 2	0.746(0.685–0.806)	2.2	0.028	0.306(0.069–0.543)	0.000	0.030(0.014–0.047)	0.000
MACE	Model 3	0.765(0.707–0.822)	2.42	0.016	0.400(0.243–0.709)	0.000	0.052(0.029–0.074)	0.000
MACE	Model 2	-	Reference	-	Reference	-	Reference	-
MACE	Model 3	-	1.33	0.184	0.436(0.201–0.672)	0.022	0.007(0.007–0.036)	0.000

Model 1: Hypertension, DM, Previous PCI, Gensini scores group, WBC, Hb, ALB, ApoA, CHOL, NT-proBNP

Model 2: Model1 + LVEF, LVEDV, LAEF

Model 3: Model2 + LACI

*DM* diabetes mellitus, *PCI* percutaneous coronary intervention, *WBC* white blood cell count, *Hb* hemoglobin, *ALB* albumin, *ApoA* Apolipoprotein A, *CHOL* total cholesterol, *NT-proBNP* N-terminal pro-B-type Natriuretic Peptid, *LVEDV* left ventricular end-diastolic volume, *LVEF* left ventricular ejection fraction, *LAEF* left atrial ejection fraction, *LACI* left atrial-ventricular coupling index

### Reproducibility analysis

Measurement reproducibility of LACI was performed for the study group by using tracings from two independent operators. The ICC was 0.982 (95% CI [0.975–0.987]), and Inter-group correlation coefficient was 0.970 (95% CI [0.960–0.980]).

## Discussion

The main findings of the present study are the following: (i) The LACI, quantified through AI-enhanced 3DE analysis, demonstrated an independent correlation with adverse outcomes in our large CAD cohort. (ii) CAD patients with elevated LACI values demonstrated significantly increased risk of MACE, especially in male patients and patients with severe lesion subgroups by Gensini scores. (iii) Through ROC analysis combined with NRI and IDI metrics, LACI emerged as a superior risk stratification tool relative to conventional echocardiographic parameters. These cumulative results establish LACI as a clinically practical tool that refined prognostic evaluation in CAD.

Extensive studies have established that CAD promoted LV structural remodeling, leading to diastolic dysfunction and increased myocardial stiffness [31]. In AMI patients, plaques may disrupt vascular tone and impair LV diastolic function [32]. Progressive LA enlargement, a hallmark of advanced CAD [33], served as a compensatory mechanism for impaired ventricular filling, resulting in disproportionate LA-LV volume ratios that elevated LACI. This atrioventricular mismatch reflected deteriorating cardiac mechanics and portended adverse cardiovascular outcomes.

While prior research focused on isolated LA or LV parameters, such as LA dimension (LAd) [16, 19], our study emphasized the prognostic significance of LA-LV coupling dynamics. During early diastole, vortex-driven blood flow augmented kinetic energy transfer and LV expansion, while diastasis optimized preload via cardiomyocyte stretching [17]. These hemodynamic interactions underscore the physiological basis for LACI ‘s prognostic value as a composite measure of LA and LV end-diastolic volumes [20–22, 34].

Although LACI has been validated for predicting HF [18] and AF [35], its application in CAD remains limited. Our findings established a significant association between elevated LACI and MACE incidence, attributable to its reflection of impaired atrial function and ventricular compliance [36, 37]. Notably, LACI surpassed traditional echocardiographic parameters in MACE prediction, supporting its potential as a novel imaging biomarker. The causal mediation analysis revealed that after adjusting for a range of important clinical confounders, the mediating role of LVDD in the association between LACI and MACE was nonsignificant. However, an association between LVDD and LACI was observed, suggesting that LVDD may act as a common correlate of both LACI and MACE rather than a critical component in the causal pathway.

While the Gensini score accurately depicts the extent of coronary atherosclerosis [23, 24], it does not fully capture the functional myocardial consequences of chronic ischemia. A elevated LACI signifies impaired atrial contractile function, which often develops as a compensatory mechanism in response to increased LV filling pressures and diastolic dysfunction—common sequelae of advanced CAD. Therefore, LACI serves as an integrated marker of the hemodynamic toll exacted by coronary disease on the left heart. Our integration of the two parameters yielded improved prognostic discrimination, as evidenced by modest but statistically significant NRI and IDI values. The incremental prognostic value of LACI suggests that combining anatomical assessment (Gensini score) with functional assessment (LACI) offers a more robust risk stratification paradigm, identifying patients who, despite a given anatomical burden, have developed more advanced functional impairment and are thus at heightened risk for future events.

Most prior LACI studies employed 2DE Simpson’s method, which lacked volumetric accuracy [8]. Although CMR offered precision, its limited accessibility rendered 3DE a more practical alternative [17]. Our AI-enhanced 3DE approach (DHM) enabled automated, reproducible measurement of LAVmin and LVEDV, streamlining LACI assessment while overcoming the limitations of manual 2D techniques.

##  Limitation

Despite demonstrating LACI ‘s prognostic value in a large CAD cohort, this study has several limitations. Firstly, the retrospective single-center design may introduce selection bias despite rigorous inclusion criteria. Furthermore, as strain parameters were not measured, the prognostic performance of LACI was evaluated against conventional echocardiographic parameters (e.g., LVEF, LAEF, and LAVI), but was not assessed against more sensitive parameters like ventricular or atrial strain. Consequently, while the improvement in risk prediction was statistically significant, its modest magnitude may constrain the interpretation of LACI’s incremental predictive value. Thirdly, subgroup analyses demonstrated nonsignificant associations in female patients, which may be attributable to the imbalanced sex distribution (69% male) or inherent biological differences. Consequently, further validation in independent cohorts is warranted. In addition, there is no precisely defined accuracy for LACI measurements obtained using DHM, but previous studies indicated that 3DE was more accurate than 2DE in measuring cardiac volume [8, 9]. And this AI-based measurement tool has been widely adopted and validated in multiple research studies, demonstrating its utility as a reliable methodological approach in the field [38]. Finally, due to the relatively short follow-up period and the consequent limited number of endpoint events, particularly the predominance of unstable angina in the composite endpoint, future studies will extend the follow-up duration and perform stratified subgroup analyses for individual MACE components.

## Conclusion

LACI, as quantified through AI-enhanced 3DE, emerges as an independent prognostic indicator for MACE in patients with CAD. This association is particularly pronounced in individuals with severe coronary lesions. LACI demonstrates superior discriminative capability relative to conventional echocardiographic parameters, suggesting its potential to enhance current risk stratification frameworks. The incorporation of LACI assessment could consequently contribute to reducing the CAD-related global burden.

## Supplementary Information

Supplementary Material 1: Supplementary Figure 1. Mediation analysis of the effect of LACI on MACE through LVDD. Model 1: no covariables adjusted. Model 2: age, sex, BMI, hypertension, DM, previous PCI, Gensini scores group, WBC, hemoglobin, ALB, ApoA, CHOL, NT-proBNP.

Supplementary Material 2: Supplementary Figure 2. ROC analysis for incremental predictive value of LACI in all CAD patients. Model 1: Hypertension, DM, Previous PCI, Gensini scores group, WBC, Hb, ALB, ApoA, CHOL, NT-proBNP. Model 2: Model 1+LVEF, LVEDV, LAEF. Model 3: Model 2+LACI.

Supplementary Material 3: Supplementary Table 1. Univariable and multivariable analysis of MACE occurrence. All continuous parameters were standardized. MACE, major adverse cardiovascular events; BMI, body mass index; BSA, body surface area; SBP, systolic blood pressure; DBP, diastolic blood pressure; DM, diabetes mellitus; PCI, percutaneous coronary intervention; WBC, white blood cell counts; PLT, platelet; HB, hemoglobin; 2D, two-dimensional; LAD, left atrial diameter; LVDd, left ventricular end-diastolic dimension; LVDs, left ventricular end-systolic dimension; RVOTD, right ventricular outflow tract diameter; LACI, left atrial-ventricular coupling index; LAEF, left atrial ejection fraction; LVEDV, left ventricular end-diastolic volume; LVESV, left ventricular end-systolic volume; LVEF, left ventricular ejection fraction; E/A, ratio of early to late diastolic peak velocities; e’, early diastolic velocity of mitral annulus; Sep e’, septal early diastolic mitral annular velocity; Lat e’, lateral early diastolic mitral annular velocity. Supplementary Table 2. Variance Inflation Factor and Tolerance. LACI, left atrial-ventricular coupling index; LVEDmass, left ventricular end-diastolic mass; LAVmin, minimal left atrial volume; LAEF, left atrial ejection fraction; LAVI, left atrial volume index; LVEDV, left ventricular end-diastolic volume; LVEF, left ventricular ejection fraction. Supplementary Table 3. The correlation between LACI and other echocardiography parameters. 2D, two-dimensional; LAD, left atrial diameter; LVDd, left ventricular end-diastolic dimension; LVDs, left ventricular end-systolic dimension; RVOTD, right ventricular outflow tract diameter; MVE, mitral valve E velocity; MVA, mitral valve A velocity; E/A, ratio of early to late diastolic peak velocities; e’, early diastolic velocity of mitral annulus; Sep e’, septal early diastolic mitral annular velocity; Lat e’, lateral early diastolic mitral annular velocity; LAVmin, minimal left atrial volume; LACI, left atrial-ventricular coupling index; LVEDmass, left ventricular end-diastolic mass; LAVmax, maximal left atrial volume; LAEF, left atrial ejection fraction; LAVI, left atrial volume index; LVEDV, left ventricular end-diastolic volume; LVESV, left ventricular end-systolic volume; LVEF, left ventricular ejection fraction. Supplementary Table 4. Mediation analysis of the effect of LACI on MACE through LVDD. Supplementary Table 5. Incremental predictive value of LACI in the overall cohort. DM, diabetes mellitus; PCI, percutaneous coronary intervention; WBC, white blood cell count; Hb, hemoglobin; ALB, albumin; ApoA, Apolipoprotein A; CHOL, total cholesterol; NT-proBNP, N-terminal pro-B-type Natriuretic Peptid; LVEDV, left ventricular end-diastolic volume; LVEF, left ventricular ejection fraction; LAEF, left atrial ejection fraction; LACI, left atrial-ventricular coupling index. Model 1: Hypertension, DM, Previous PCI, Gensini scores group, WBC, Hb, ALB, ApoA, CHOL, NT-proBNP. Model 2: Model 1+LVEF, LVEDV, LAEF. Model 3: Model 2+LACI.

## Data Availability

The datasets generated for and analyzed in the study are not publicly available due to China Medical University’s privacy policy but are available from the corresponding author upon reasonable request.

## Abbreviations

AI: Artificial intelligence
AF: Atrial fibrillation
BMI: Body mass index
CMR: Cardiac magnetic resonance
CAD: Coronary artery disease
CAD: Coronary Heart Disease
DM: Diabetes mellitus
DBP: Diastolic blood pressure
DHM: Dynamic Heart Model
HFrEF: Heart failure with reduced ejection fraction
IDI: Integrated classification improvement
ICC: Intraclass Correlation Coefficient
LAd: Left atrial diameter
LACI: Left atrial-ventricular coupling index
LAVmin: Minimal left atrial volume
LA: Left atrium
LV: Left ventricle
LVEDV: Left ventricular end-diastolic volume
MACE: Major adverse cardiovascular events
NRI: Net reclassification improvement
PCI: Percutaneous coronary intervention
SBP: Systolic blood pressure
3DE: Three-dimensional echocardiograph
