# Supplementary figures and images for: Artificial intelligence-enhanced three-dimensional echocardiography reveals left atrial-ventricular coupling index as a novel prognostic marker in coronary artery disease
Source: BMC Cardiovasc Disord. 2025 Dec 3;25:859. doi: 10.1186/s12872-025-05341-z (PMC12676787; doi:10.1186/s12872-025-05341-z)

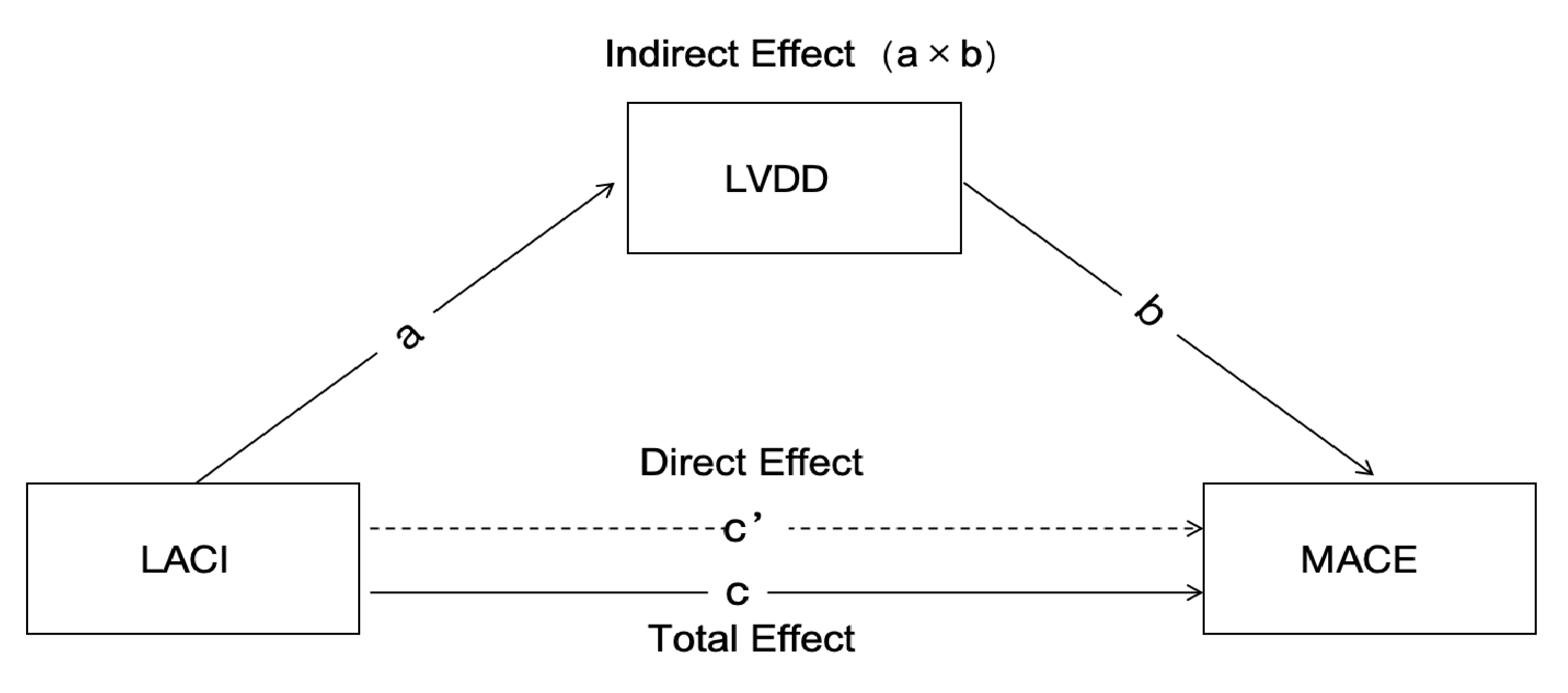

Supplement: Supplementary file 1 — Supplementary Material 1: Supplementary Figure 1. Mediation analysis of the effect of LACI on MACE through LVDD. Model 1: no covariables adjusted. Model 2: age, sex, BMI, hypertension, DM, previous PCI, Gensini scores group, WBC, hemoglobin, ALB, ApoA, CHOL, NT-proBNP. [file 12872_2025_5341_MOESM1_ESM.tif]

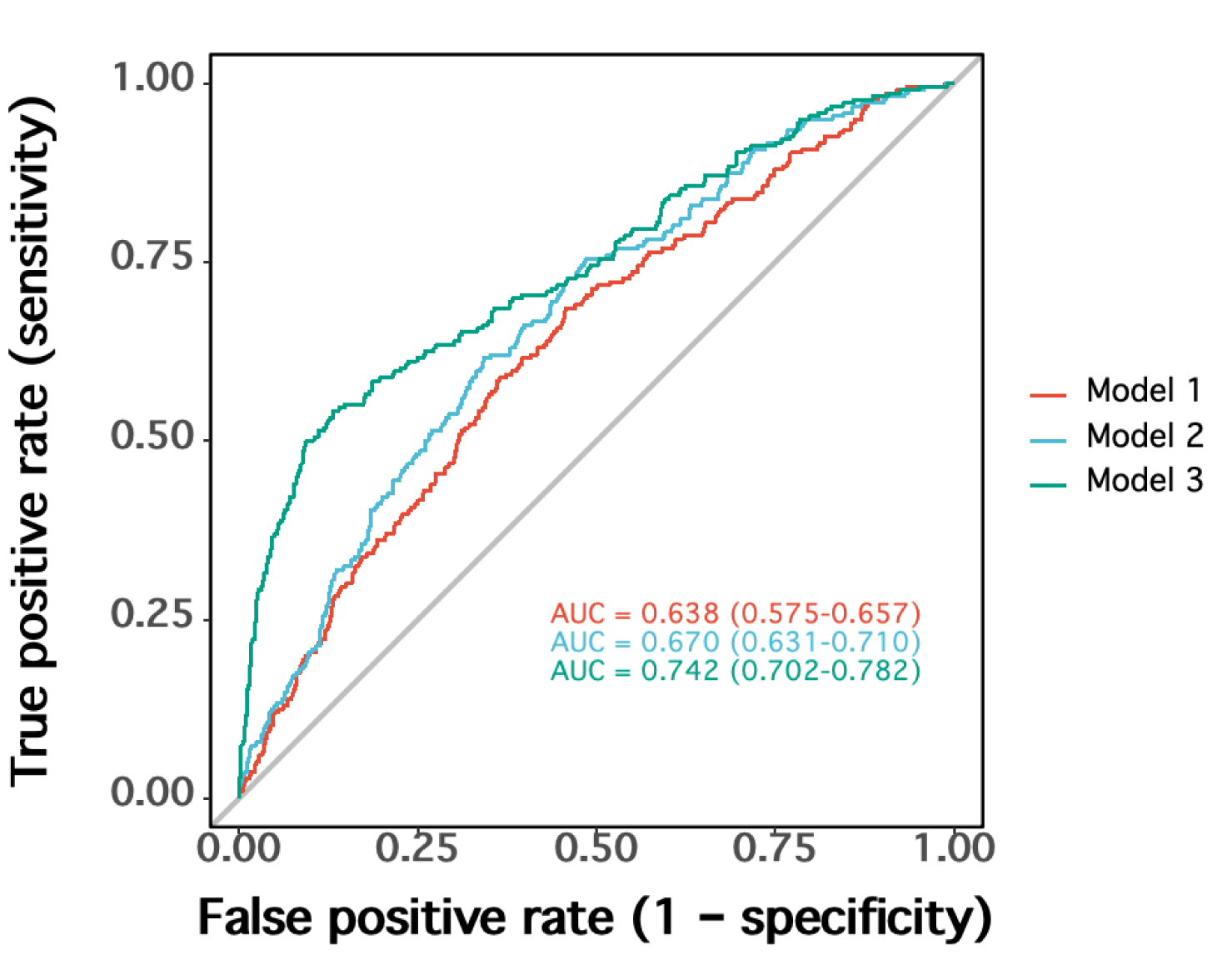

Supplement: Supplementary file 2 — Supplementary Material 2: Supplementary Figure 2. ROC analysis for incremental predictive value of LACI in all CAD patients. Model 1: Hypertension, DM, Previous PCI, Gensini scores group, WBC, Hb, ALB, ApoA, CHOL, NT-proBNP. Model 2: Model 1+LVEF, LVEDV, LAEF. Model 3: Model 2+LACI. [file 12872_2025_5341_MOESM2_ESM.tif]
