# Supplementary material for: Artificial intelligence-enhanced three-dimensional echocardiography reveals left atrial-ventricular coupling index as a novel prognostic marker in coronary artery disease
Source: BMC Cardiovasc Disord. 2025 Dec 3;25:859. doi: 10.1186/s12872-025-05341-z (PMC12676787; doi:10.1186/s12872-025-05341-z)
Supplement: Supplementary file 3 — Supplementary Material 3: Supplementary Table 1. Univariable and multivariable analysis of MACE occurrence. All continuous parameters were standardized. MACE, major adverse cardiovascular events; BMI, body mass index; BSA, body surface area; SBP, systolic blood pressure; DBP, diastolic blood pressure; DM, diabetes mellitus; PCI, percutaneous coronary intervention; WBC, white blood cell counts; PLT, platelet; HB, hemoglobin; 2D, two-dimensional; LAD, left atrial diameter; LVDd, left ventricular end-diastolic dimension; LVDs, left ventricular end-systolic dimension; RVOTD, right ventricular outflow tract diameter; LACI, left atrial-ventricular coupling index; LAEF, left atrial ejection fraction; LVEDV, left ventricular end-diastolic volume; LVESV, left ventricular end-systolic volume; LVEF, left ventricular ejection fraction; E/A, ratio of early to late diastolic peak velocities; e’, early diastolic velocity of mitral annulus; Sep e’, septal early diastolic mitral annular velocity; Lat e’, lateral early diastolic mitral annular velocity. Supplementary Table 2. Variance Inflation Factor and Tolerance. LACI, left atrial-ventricular coupling index; LVEDmass, left ventricular end-diastolic mass; LAVmin, minimal left atrial volume; LAEF, left atrial ejection fraction; LAVI, left atrial volume index; LVEDV, left ventricular end-diastolic volume; LVEF, left ventricular ejection fraction. Supplementary Table 3. The correlation between LACI and other echocardiography parameters. 2D, two-dimensional; LAD, left atrial diameter; LVDd, left ventricular end-diastolic dimension; LVDs, left ventricular end-systolic dimension; RVOTD, right ventricular outflow tract diameter; MVE, mitral valve E velocity; MVA, mitral valve A velocity; E/A, ratio of early to late diastolic peak velocities; e’, early diastolic velocity of mitral annulus; Sep e’, septal early diastolic mitral annular velocity; Lat e’, lateral early diastolic mitral annular velocity; LAVmin, minimal left atrial volume; LAC [file 12872_2025_5341_MOESM3_ESM.docx]

**Supplementary table 1 : Univariable and multivariable Analysis of MACE occurrence**

| **Characteristic** | **Univariable** | | | **Multivariable** | | |
| --- | --- | --- | --- | --- | --- | --- |
|  | **HR** | **95% CI** | ***p*-value** | **HR** | **95% CI** | ***p*-value** |
| **Sex (female)** | 1.07 | 0.80, 1.42 | 0.658 |  |  |  |
| **Age group** |  |  |  |  |  |  |
| [26,51.2) | reference | reference |  |  |  |  |
| [51.2,63.3) | 0.76 | 0.49, 1.20 | 0.239 |  |  |  |
| [63.3,74.2) | 0.99 | 0.64, 1.52 | 0.963 |  |  |  |
| [74.2,90] | 1.07 | 0.65, 1.78 | 0.782 |  |  |  |
| **BMI** | 1.03 | 0.90, 1.18 | 0.704 |  |  |  |
| **BSA** | 1.01 | 0.88, 1.15 | 0.935 |  |  |  |
| **SBP** | 0.97 | 0.85, 1.11 | 0.707 |  |  |  |
| **DBP** | 0.93 | 0.81, 1.06 | 0.290 |  |  |  |
| **Previous smoke** | 1.08 | 0.83, 1.41 | 0.563 |  |  |  |
| **Previous drink** | 0.98 | 0.68, 1.40 | 0.898 |  |  |  |
| **Hypertension** | 1.50 | 1.11, 2.02 | 0.008 | 1.42 | 1.04, 1.95 | 0.029 |
| **DM** | 1.55 | 1.18, 2.02 | 0.001 | 1.30 | 0.98, 1.72 | 0.071 |
| **AF** | 1.48 | 0.89, 2.46 | 0.133 |  |  |  |
| **Previous MI** | 1.37 | 1.03, 1.82 | 0.030 | 1.32 | 0.98, 1.76 | 0.063 |
| **Previous PCI** | 1.66 | 1.26, 2.17 | <0.001 | 1.47 | 1.11, 1.94 | 0.007 |
| **Gensini group** |  |  |  |  |  |  |
| mild lesion group | reference | reference |  | reference | reference |  |
| moderate lesion group | 1.51 | 0.82, 2.80 | 0.187 | 1.38 | 0.74, 2.56 | 0.313 |
| severe lesion group | 2.41 | 1.34, 4.36 | 0.003 | 1.97 | 1.08, 3.61 | 0.028 |
| **Hypertension medication** | 0.79 | 0.34, 1.85 | 0.593 |  |  |  |
| **Lipid-lowering medication** | 1.38 | 0.37, 5.16 | 0.635 |  |  |  |
| **WBC** | 1.13 | 1.07, 1.20 | <0.001 | 1.15 | 1.09, 1.22 | <0.001 |
| **PLT** | 1.03 | 0.90, 1.17 | 0.660 |  |  |  |
| **HB** | 0.86 | 0.75, 0.98 | 0.025 | 0.90 | 0.76, 1.06 | 0.205 |
| **ALB** | 0.85 | 0.74, 0.97 | 0.018 | 0.92 | 0.79, 1.08 | 0.309 |
| **CHOL** | 0.84 | 0.74, 0.97 | 0.014 | 0.91 | 0.78, 1.05 | 0.208 |
| **HDL** | 0.85 | 0.73, 0.98 | 0.026 | 0.79 | 0.60, 1.05 | 0.104 |
| **ApoA** | 0.86 | 0.75, 0.99 | 0.036 | 0.92 | 0.78,1.08 | 0.312 |
| **ApoB** | 0.88 | 0.77, 1.01 | 0.061 |  |  |  |
| **NT-pro BNP** | 1.32 | 1.18, 1.46 | <0.001 | 1.27 | 1.14, 1.42 | <0.001 |
| **LAD-2D** | 1.26 | 1.11, 1.44 | <0.001 | 1.08 | 0.92, 1.28 | 0.346 |
| **LVDd-2D** | 1.23 | 1.09, 1.39 | <0.001 | 1.08 | 0.84, 1.39 | 0.549 |
| **LVDs-2D** | 1.24 | 1.10, 1.39 | <0.001 | 1.07 | 0.84, 1.38 | 0.567 |
| **RVOTD-2D** | 0.94 | 0.82, 1.07 | 0.344 | 0.89 | 0.78, 1.02 | 0.104 |
| **MVE** | 1.14 | 1.00, 1.30 | 0.051 |  |  |  |
| **MVA** | 1.00 | 0.87, 1.14 | 0.975 |  |  |  |
| **Sep e’** | 0.93 | 0.81, 1.06 | 0.280 |  |  |  |
| **Lat e’** | 0.98 | 0.86, 1.12 | 0.748 |  |  |  |
| **E/A-2D** | 1.06 | 0.96, 1.16 | 0.266 |  |  |  |
| **E/e’-2D** | 1.17 | 1.06, 1.30 | 0.002 | 0.94 | 0.80, 1.11 | 0.497 |
| **LAVmin-3D** | 1.30 | 1.17, 1.44 | <0.001 | 1.25 | 1.13, 1.39 | <0.001 |
| **LACI** | 1.27 | 1.14, 1.42 | <0.001 | 1.49 | 1.28, 1.74 | <0.001 |
| **LAVI-3D** | 1.12 | 0.99, 1.27 | 0.066 |  |  |  |
| **LAVmax-3D** | 1.13 | 0.99, 1.28 | 0.063 |  |  |  |
| **LAEF-3D** | 0.72 | 0.64, 0.81 | <0.001 | 0.72 | 0.63, 0.81 | <0.001 |
| **LVEDmass-3D** | 1.13 | 1.00, 1.29 | 0.058 |  |  |  |
| **LVEDV-3D** | 1.19 | 1.06, 1.34 | 0.003 | 1.97 | 1.16, 3.37 | 0.012 |
| **LVESV-3D** | 1.21 | 1.09, 1.34 | <0.001 | 0.60 | 0.31, 1.17 | 0.137 |
| **LVEF-3D** | 0.81 | 0.71, 0.91 | <0.001 | 0.73 | 0.52, 1.03 | 0.077 |
| HR = Hazard Ratio, CI = Confidence Interval.  All continuous parameters were standardized. MACE, major adverse cardiovascular events; BMI, body mass index; BSA, body surface area; SBP, systolic blood pressure; DBP, diastolic blood pressure; DM, diabetes mellitus; PCI, percutaneous coronary intervention; WBC, white blood cell counts; PLT, platelet; HB, hemoglobin; 2D, two-dimensional; LAD, left atrial diameter; LVDd, left ventricular end-diastolic dimension; LVDs, left ventricular end-systolic dimension; RVOTD, right ventricular outflow tract diameter; LACI, left atrial-ventricular coupling index; LAEF, left atrial ejection fraction; LVEDV, left ventricular end-diastolic volume; LVESV, left ventricular end-systolic volume; LVEF, left ventricular ejection fraction; E/A, ratio of early to late diastolic peak velocities (or E-wave to A-wave ratio); Sep e’, septal early diastolic mitral annular velocity; Lat e’, lateral early diastolic mitral annular velocity. | | | | | | |

**Supplementary table 2: Variance Inflation Factor and Tolerance**

| Term | VIF | VIF_CI_low | VIF_CI_high | SE_factor | Tolerance | Tolerance_CI_low | Tolerance_CI_high |
| --- | --- | --- | --- | --- | --- | --- | --- |
| LACI | 12.371225 | 11.054233 | 13.860728 | 3.517275 | 0.08083274 | 0.07214628 | 0.09046309 |
| LAVI | 4.972485 | 4.478647 | 5.536429 | 2.229907 | 0.20110671 | 0.18062184 | 0.22328173 |
| LAVmin | 16.789704 | 14.981242 | 18.832090 | 4.097524 | 0.05956031 | 0.05310085 | 0.06675014 |
| LAEF | 4.763631 | 4.293044 | 5.301467 | 2.182574 | 0.20992389 | 0.18862702 | 0.23293496 |
| LVEDV | 9.552485 | 8.549044 | 10.689308 | 3.090710 | 0.10468480 | 0.09355143 | 0.11697215 |
| LVEF | 1.569964 | 1.456793 | 1.711173 | 1.252982 | 0.63695740 | 0.58439461 | 0.68643932 |
| LVEDmass | 3.464845 | 3.138925 | 3.840428 | 1.861410 | 0.28861316 | 0.26038763 | 0.31858041 |

LACI, left atrial-ventricular coupling index; LVEDmass, left ventricular end-diastolic mass; LAVmin, minimal left atrial volume ; LAEF, left atrial ejection fraction; LAVI, left atrial volume index; LVEDV, left ventricular end-diastolic volume; LVEF, left ventricular ejection fraction.

**Supplementary table 3: The correlation between LACI and other echocardiography parameters**

| **Parameter1** | | **Parameter2** | | **r** | | ***p*** | |
| --- | --- | --- | --- | --- | --- | --- | --- |
| **LAD-2D** | | **LACI** | | 0.388042525 | | <0.001 | |
| **LVDd-2D** | | **LACI** | | 0.053582993 | | 0.078 | |
| **RVOTD-2D** | | **LACI** | | 0.045087593 | | 0.138 | |
| **MVE** | | **LACI** | | 0.153379408 | | <0.001 | |
| **MVA** | | **LACI** | | 0.003297129 | | 0.914 | |
| **Sep e’, cm/s** | | **LACI** | | -0.148192233 | | <0.001 | |
| **Lat e’, cm/s** | | **LACI** | | -0.154355175 | | <0.001 | |
| **E/A-2D** | | **LACI** | | 0.119068445 | | <0.001 | |
| **E/e’-2D** | | **LACI** | | 0.280110840 | | <0.001 | |
| **LACI** | | **LVEDmass** | | 0.032965950 | | 0.278 | |
| **LACI** | | **LAVmax** | | 0.540770293 | | <0.001 | |
| **LACI** | | **LAEF** | | -0.624099308 | | <0.001 | |
| **LACI** | | **LAVI** | | 0.590180681 | | <0.001 | |
| **LACI** | | **LVESV** | | -0.015953072 | | 0.600 | |
| **LACI** | | **LVEF** | | 0.030792787 | | 0.311 | |
| **LAVmin** | | **LVEDV** | | 0.569291652 | | <0.001 | |
| 2D, two-dimensional; LAD, left atrial diameter; LVDd, left ventricular end-diastolic dimension; LVDs, left ventricular end-systolic dimension; RVOTD, right ventricular outflow tract diameter; MVE,mitral valve E velocity; MVA, mitral valve A velocity; E/A, ratio of early to late diastolic peak velocities (or E-wave to A-wave ratio); Sep e’, septal early diastolic mitral annular velocity; Lat e’, lateral early diastolic mitral annular velocity; LAVmin, minimal left atrial volume; LACI, left atrial-ventricular coupling index; LVEDmass, left ventricular end-diastolic mass; LAVmax, maximal left atrial volume ; LAEF, left atrial ejection fraction; LAVI, left atrial volume index; LVEDV, left ventricular end-diastolic volume; LVESV, left ventricular end-systolic volume; LVEF, left ventricular ejection fraction. | | | | | | | |

**Supplementary table 4: Mediation analysis of the effect of LACI on MACE through LVDD**

| Model | Effect Type | Coefficient (95% CI) | P Value | Proportion Mediated, %  (95% CI) |
| --- | --- | --- | --- | --- |
| Unadjusted | Total | -2.82 (-4.40, -1.32) | <0.001 | — |
|  | Indirect | -0.54 (-1.04, -0.08) | 0.028 | — |
|  | Direct | -2.29 (-3.83, -0.80) | 0.004 | — |
|  | Overall | — | — | 18.0 (3.2, 46.0) |
| Adjusted* | Total | -56.92 (-147.39, -10.96) | 0.004 | — |
|  | Indirect | -6.46 (-10.87, 2.93) | 0.484 | — |
|  | Direct | -50.46 (-124.98, -9.97) | <0.001 | — |
|  | Overall | — | — | 3.4 (-10.0, 29.2) |
| *Adjusted for age, sex, BMI, hypertension, diabetes mellitus (DM), previous PCI, Gensini scores group, white blood cell count (WBC), hemoglobin, albumin (ALB), apolipoprotein A (ApoA), cholesterol (CHOL), and NT-proBNP. | | | | |

| **Outcome** | **Model** | **AUC 95% CI** | **Delong Test** | | **NRI** | | **IDI** | |
| --- | --- | --- | --- | --- | --- | --- | --- | --- |
|  |  |  | **Z score** | ***p*-value** | **95% CI** | ***p*-value** | **95% CI** | ***p*-value** |
| **Mace** | **Model1** | 0.638(0.575 - 0.657) | **Reference** | - | **Reference** | - | **Reference** | - |
| Mace | Model2 | 0.670(0.631-0.710) | 2.459 | 0.014 | 0.276(0.129-0.424) | 0.000 | 0.020(0.009-0.031) | 0.000 |
| Mace | Model3 | 0.742(0.702 - 0.782) | 4.118 | < 0.001 | 0.341(0.194-0.489) | 0.000 | 0.033(0.020-0.047) | 0.000 |
| **Mace** | **Model2** | - | **Reference** | - | **Reference** | - | **Reference** | - |
| Mace | Model3 | - | 3.049 | 0.002 | 0.189(0.406-0.338) | 0.013 | 0.004(0.006-0.020) | 0.000 |

**Supplementary table 5: Incremental predictive value of LACI in the overall cohort**

DM, diabetes mellitus; PCI, percutaneous coronary intervention; WBC, white blood cell count; Hb, hemoglobin; ALB, albumin; ApoA, Apolipoprotein A; CHOL, total cholesterol; NT-proBNP, N-terminal pro-B-type Natriuretic Peptid; LVEDV, left ventricular end-diastolic volume; LVEF, left ventricular ejection fraction; LAEF, left atrial ejection fraction; LACI, left atrial-ventricular coupling index.

**Model 1: Hypertension, DM, Previous PCI, Gensini scores group, WBC, Hb, ALB, ApoA, CHOL, NT-proBNP**

**Model 2: Model 1+LVEF, LVEDV, LAEF**

**Model 3: Model 2+LACI**
